# ESBL and *Amp*C β-Lactamase Encoding Genes in *E. coli* From Pig and Pig Farm Workers in Vietnam and Their Association With Mobile Genetic Elements

**DOI:** 10.3389/fmicb.2021.629139

**Published:** 2021-03-11

**Authors:** Yaovi Mahuton Gildas Hounmanou, Valeria Bortolaia, Son Thi Thanh Dang, Duong Truong, John E. Olsen, Anders Dalsgaard

**Affiliations:** ^1^Department of Veterinary and Animal Sciences, University of Copenhagen, Frederiksberg, Denmark; ^2^Research Group for Genomic Epidemiology, National Food Institute, Technical University of Denmark, Kgs. Lyngby, Denmark; ^3^Veterinary Hygiene Department, National Institute of Veterinary Research, Hanoi, Vietnam; ^4^School of Chemical and Biomedical Engineering, Nanyang Technological University, Singapore, Singapore

**Keywords:** ESBL, mobile genetic element, genomics, one health, antimicrobial resistance, pigs, workers, Vietnam

## Abstract

Animals are considered important sources of ESBL/AmpC-producing bacteria in humans. We analyzed indications of transfer of ESBL/AmpC genes between pigs and pig farmers in Vietnam by analyzing whole genome sequences of 114 ESBL/AmpC-producing *E. coli* isolated from the two hosts, and performed conjugation experiments and plasmid profiling to confirm that such transfer could have happened. ESBL-encoding genes detected in pigs and pig farmers included *bla*_CTX–M-55_, *bla*_CTX-M-27_, *bla*_CTX-M-65_, *bla*_CTX-M-15_, *bla*_CTX-M-14_, *bla*_CTX-M-3_, *bla*_CTX-M-24_, and *bla*_CARB-2_, and AmpC β-lactamases included *bla*_CMY-2_, *bla*_DHA-1_, and *bla*_CMY-42_. The most frequent ESBL gene, *bla*_CTX-M-55_, was carried on plasmid with replicons types IncF, IncX, IncH, IncN, IncR, and IncP. The insertion transposases downstream of the *bla*_CTX-M-55_ gene were different in plasmids carried by different strains. The second most detected gene, *bla*_CTX-M-27_, is found in a stable genetic arrangement with the same flanking transposons seen across strains, and the gene was located on similar conjugal IncF plasmid types, suggesting a horizontal spread of these plasmids. In three strains, we observed a novel *bla*_CTX-M-27_ harboring IncF type of plasmid which had not been reported before. Its closest reference in NCBI was the non-ESBL *Salmonella* Typhimurium plasmid pB71 that might have experienced an insertion of *bla*_CTX-M-27_. Our data also point to an emergence of plasmids co-carrying ESBL genes, *mcr* genes, quinolones and other antimicrobials resistance determinants, and such plasmids require special attention. Plasmids phylogeny confirmed that the *bla*_CTX-M-55_ encoding plasmids varied considerably, while those encoding *bla*_CTX-M-27_ were closely related. Plasmids harboring both ESBL genes were confirmed to be conjugative and not to differ in transfer efficacy. The isolates carrying the plasmids, even those with plasmids of similar types, showed wide genetic variation with high number of SNPs, suggesting horizontal spread of plasmids into different clonal lines. Their virulence profiles did not confirm to known pathotypes, suggesting that unrelated commensals are a main reservoir for ESBL and AmpC β-lactamases in both humans and pigs. Overall, despite evidence of transferability of plasmids in the analyzed strains, our findings do not support that ESBL-producing *E. coli* from pigs or their ESBL/AmpC encoding plasmids are commonly spread to workers in close contact with the animals.

## Introduction

Extended-spectrum β-lactamases (ESBLs) and AmpC β-lactamases are the most widespread mechanisms of cephalosporin resistance in *E. coli* in animals and humans worldwide, including the Asia Pacific region (Jean and Hsueh, 2017; Naas et al., 2017). Some of the ESBLs and AmpC β-lactamases described in *E. coli* have spread globally in the last two decades, including CMY-2, which is the most common AmpC enzyme (Naas et al., 2017), and CTX-M, which is the most widespread ESBL type (Jean and Hsueh, 2017); however, there are marked differences in the CTX-M variants occurring across sources and geographical areas. In Vietnam, CTX-M-1 group enzymes including CTX-M-15 and CTX-M-55, and CTX-M-9 group enzymes including CTX-M-14, CTX-M-27, and CTX-M-65 have been described in *E. coli* from diseased and healthy humans, chickens, pigs, meat, and vegetables (Bui et al., 2015; Zurfluh et al., 2015; Nguyen et al., 2016; Hoang et al., 2017; Jean and Hsueh, 2017).

In most cases, ESBL/AmpC-encoding genes are located on mobile genetic element, and many are plasmid-mediated and transferable between bacteria of different species. Studies from Europe have shown that farmers share highly related and even identical ESBL/AmpC *E. coli* and/or ESBL/AmpC plasmids with their livestock (Madec et al., 2017). It is likely that this risk is enhanced within countries with higher level of animal to human contact due to low hygienic standards in their livestock production, such as in pig production in Vietnam (Hoang et al., 2017), but this remains to be further investigated.

Knowledge and control of ESBL and AmpC in *E. coli* from animals is crucial to preserve the effect of medically important antimicrobials such as cephalosporins. Therefore, ESBL/AmpC monitoring in *E. coli* from animals and food has been mandatory in EU countries since 2014 (Commission implementing Decision 2013/652/EU) and such monitoring is a suggested focus area by the World Health Organization in low-and middle-income countries (The ESBL Ec Tricycle AMR surveillance project)^1^.

In Vietnam, knowledge on the ESBL/AmpC variants in *E. coli* from pigs and pig farmers is limited, and thorough characterization of the genetic location of the ESBL/AmpC-encoding genes in *E. coli* is lacking. Moreover, most genomic studies that address transmission of resistance genes between animals and humans rely on tracking clonally evolving lineages excluding extrachromosomal plasmids despite plasmids being the primary carriers of antibiotic resistance genes across the pathogens (David et al., 2020).

The objective of this study was therefore to characterize ESBL/AmpC genes in *E. coli* in pigs and pig farmers in northern Vietnam and determine their location and context on mobile genetic elements to estimate the level of transfer between pigs and humans. Information from this study illustrates the role of different genetic elements in the spread of ESBL/AmpC genes between human and animal hosts.

## Materials and Methods

### Isolates and Genome Collections

In a previous study, cefotaxime-resistant *E. coli* collected in 2015 from pigs and farm workers in Thai Binh and Hanoi provinces in Vietnam were characterized (Dang et al., 2018). Forty of these cefotaxime-resistant *E. coli* was selected for whole genome sequencing by stratified random selection of isolates. At least one isolate per cephalosporin resistance profile compatible with ESBL/AmpC production was included. When the same resistance profile was present in more than one isolate, a number of isolates proportional to the frequency of that phenotype within the overall collection of isolates was selected. Choice of isolates within specific resistance profiles was random, in that, each isolate was assigned a consecutive number and random numbers were generated by using online freely available software^2^. Since we aimed at having pig and human isolates equally represented within each profile, the numbers were randomly generated until this criterion was satisfied. Only in one case, four isolates from humans and two isolates from pigs (instead of 3 and 3) were selected since the third isolate originating from pigs was no longer available.

Additionally, 74 genomes of ESBL/AmpC producing *E. coli* isolated in 2018 from pigs and pig farm workers in Bac Ninh province, Vietnam were retracted from a sequence collection submitted to the European Nucleotide Archives under the project accession number PRJEB37980. Thus in total 114 genomes were included in the study.

All 114 strains were previously subjected to antimicrobial susceptibility testing, and results are available from Dang et al. (2018) and a recently submitted manuscript (Duong et al., under review).

### Whole Genome Sequencing, Genome Assembly, and Genome-Characterization

Genomic DNA was extracted from the 40 *E. coli* isolates from 2015 using an Invitrogen Easy-DNA Kit (Invitrogen, Carlsbad, CA, United States) and DNA concentration was determined using the Qubit dsDNA BR assay kit (Invitrogen). The genomic DNA was prepared for Illumina pair-end sequencing using the Illumina (Illumina, Inc., San Diego, CA) NexteraXT^®^ Guide 14 150319425031942 following the protocol revision C. The libraries were sequenced using an Illumina MiSeq platform. Raw sequence data were submitted to the European Nucleotide Archive (ENA) under study accession no: PRJEB30991. Along with the 2018 genomes retracted from ENA under the project accession PRJEB37980., the raw reads of all the 114 strains were *de novo* assembled using SPAdes algorithm for *de novo* short reads assembly (Bankevich et al., 2012). Assembled sequences were analyzed using the CGE tools^3^ including MLST finder to determine *E. coli* multi-locus sequence types (MLST), ResFinder 3.1 for detection of genes and chromosomal mutations mediating AMR, VirulenceFinder 2.0 with default settings for detection of virulence genes, PlasmidFinder for detection of plasmid replicons, and pMLST for further subtyping of specific plasmids.

### Analysis of Contigs Harboring ESBL/AmpC-Encoding Genes

In all genomes, contigs harboring ESBL/AmpC-encoding genes were analyzed in detail to gain insights into the genetic context of the ESBL/AmpC-encoding genes. First, nucleotide BLAST at NCBI was used to determine the level of identity of the contig with publicly available sequences. Then, Open Reading Frames (ORFs) were predicted and annotated using Artemis software version 8 (Carver et al., 2012), and each predicted protein was compared against the all-protein database at NCBI using BlastP. ISfinder was used for identification of insertion sequences (IS) (Siguier et al., 2006). Comparisons of contigs harboring identical ESBL/AmpC-encoding genes were performed using CLC genomics workbench v.8. Furthermore, the Artemis comparative tool (Carver et al., 2005) was used to perform in-depth comparative genomics between ESBL-hosting contigs of our samples and their closest references from NCBI.

### Plasmids Reconstruction and Characterization

To retract plasmid components from the short illumina reads for downstream analyses, the raw reads of all genomes with and without plasmid replicons were analyzed with PlasmidSpades (Bankevich et al., 2012). The predicted plasmids were then used as input files in PlasmidFinder to detect their replicon types and analyzed in ResFinder to detect antimicrobial resistance genes found on these plasmids including ESBL/AmpC encoding genes as well as co-occurrence of the plasmid-mediated colistin resistance *mcr* genes and more. Additionally, the concatenated plasmid component files were analyzed in Bacmet (Pal et al., 2014) for experimentally confirmed metal and biocide tolerance genes co-occurring on the ESBL/AmpC carrying plasmids. With a two-two-table from STATCALC in EPIINFO v.7.2, we assessed the association of metal and biocide resistance genes co-occurring with β-lactamases on the same predicted plasmids. The reconstructed plasmids were also annotated in RAST (Overbeek et al., 2014) and visualized with CLC Genomics workbench v8 and displayed as graphics for selected samples.

### Plasmid Profiling

To confirm the presence and the size of predicted plasmids in the strains, a plasmid profiling was carried out where plasmid DNA was extracted according to Kado and Liu (1981). The purified plasmid DNA was separated on a 0.8% (W/V) agarose gel for 3.5 h at 150 V and stained with 0.5 μg ml^–1^ ethidium bromide. The approximate molecular weight of each plasmid was determined by comparison with two reference *E. coli* strains, 39R861 (Threlfall et al., 1986) and V517 (Macrina et al., 1978), containing multiple reference plasmids.

### Diversity of Plasmids Harboring the Predominant ESBL Genes *bla*_CTX-M-55_ and *bla*_CTX-M-27_

Due to the fact that the ESBL/AmpC contigs were of highly variable sizes and also do not represent the whole plasmid, the predicted plasmid components were annotated using Prokka v1.13.3 (Seemann, 2014) and used for comparative analyses. Two pan-genome analyses were performed using roary v3.12 (Page et al., 2015) across the annotated gff files of predicted plasmids harboring *bla*_CTX-M-55_ and for those harboring *bla*_CTX-M-27_ to identify the number of shared genes among the plasmids. For better representation of the gene contents of the plasmids, only plasmid components with the size above 40 kb were included in these analyses making 18 plasmid components in each pan-genome.

A single nucleotide polymorphism analysis was not appropriate due to the small fraction of nucleotides representing the core-genome in the analyzed plasmids; therefore, the phylogenetic analysis was performed using nucleotides alignment method. This analysis included all predicted plasmids harboring the two *bla*_CTX-M-55_ and *bla*_CTX-M-27_), and DNA sequences were aligned using the Clustal Omega multiple sequence alignment tool^4^. The aligned files were converted to MEGA file format then used as input to construct a neighbor-joining phylogenetic tree in MEGAx (Kumar et al., 2018) using the bootstrap method with 500 replicates. The final trees were visualized in iTOL (Letunic and Bork, 2016) and rooted with the shortest plasmids in each group.

### Genetic Diversity of the *E. coli* Isolates

For the overall population analysis of the *E. coli* genomes, the whole genome sequences were analyzed in CSIPhylogeny for core-genome phylogenetic analysis using the genome of *E. coli* K12 as reference and the resulting tree was visualized in iTOL (Letunic and Bork, 2016). In this analysis, the pig isolate 51A3 obtained in 2015 containing only the narrow-spectrum β-lactamase *bla*_*TEM*–1A_ was not included.

### Conjugation Experiment

To experimentally investigate the transferability of the plasmids harboring the *bla*_CTX-M-27_ and *bla*_CTX-M-55_ genes, a conjugation experiment was conducted. We randomly selected four strains to serve as donors including EC224 (IncFII) and EC170 (IncFIB) representing *bla*_CTX-M-27_, while *bla*_CTX-M-55_ was represented by strains EC297 (IncFIB) and EC116 (IncN). The recipient strain in all experiments was *E. coli* J53-1 encoding a chromosomally located rifampicin resistance gene. The conjugation experiment was performed as previously described (Møller et al., 2017). Briefly, the donor and recipient strains were grown in Luria-Bertani (LB) broth (Sigma, Copenhagen, Denmark) with shaking (180 rpm) at 37°C to exponential phase (OD600 = 0.5). Conjugation was performed by mixing donor and recipient strains in a 1:1 ratio on filter papers (0.22 μM, Millipore, Copenhagen, Denmark) placed on LB agar plates (Becton, Dickinson, Albertslund, Denmark) at 37°C for 1 and 6 h. The bacterial material was washed from the filters using isotonic NaCl and plated on LB agar plates containing either 2 mg L^–1^ cefotaxime (to quantify donor + transconjugants), 50 mg L^–1^ rifampicin (to quantify doner and transconjugants), or 50 mg L^–1^ rifampicin and 2 mg L^–1^ cefotaxime (to quantify transconjugants) and incubated overnight at 37°C. The conjugation experiments were performed with two biological replicates and two technical replicates each. Selected colonies of the transconjugants were subjected to plasmid profiling using the method described above to confirm the transfer of the ESBL-gene encoding plasmids from the donors. The conjugation frequency of each strain was calculated as the number of transconjugants divided by the number of donors. The results are presented as means cfu. mL^–1^ ± *SD*.

## Results

### ESBL/AmpC Genes Pool

The ESBL-encoding genes detected in the *E. coli* isolates from pigs from 2015 and 2018, were *bla*_CTX-M-55_ (*n* = 28), *bla*_CTX-M-27_ (*n* = 12), *bla*_CTX-M-65_ (*n* = 4), *bla*_CTX-M-15_ (*n* = 4), and *bla*_CTX-M-14_ (*n* = 10). The AmpC β-lactamase-encoding *bla*_CMY-2_ was detected in three isolates and *bla*_DHA-1_ was present in one isolate (Supplementary Table S1). One isolate (51A3) did not yield any ESBL/AmpC-encoding gene and was not investigated further. One pig isolate co-carried *bla*_CTX-M-14_ together with the AmpC gene *bla*_DHA-1_ (Supplementary Table S1). In human isolates, the ESBL-encoding genes detected were *bla*_CTX-M-27_ (*n* = 24), *bla*_CTX-M-55_ (*n* = 12), *bla*_CTX-M-15_ (*n* = 7), *bla*_CTX-M-14_ (*n* = 5), *bla*_CTX-M-65_ (1), and the AmpC β-lactamase-encoding genes were *bla*_CMY-42_ (*n* = 1) and *bla*_CMY-2_ (*n* = 3). In addition, *bla*_CTX-M-3_ and *bla*_CTX-M-24_ were detected together in one human isolate (Supplementary Table S1). Co-occurrence of *bla*_CTX-M-27_ with AmpC genes *bla*_DHA-1_ and *bla*_CMY-2_ were detected in one strain each (Supplementary Table S1). Two 2018 strains from human samples carried *bla*_CARB-2_ in addition to *bla*_CTX-M-55_.

### Genetic Context of ESBL/AmpC Genes

#### bla_CTX-M-55_

**FIGURE 1:**
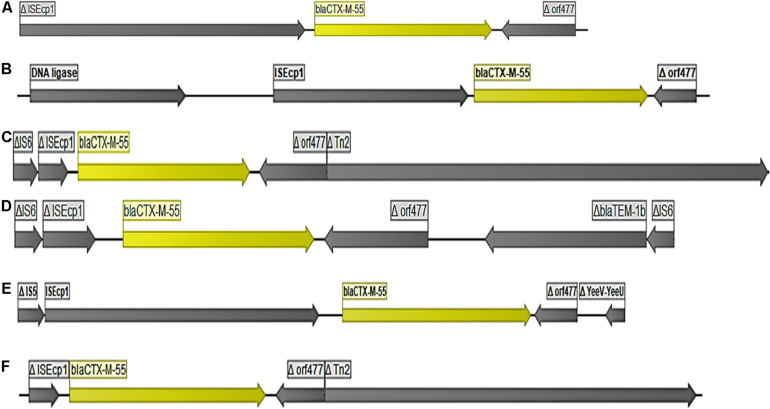
Organization of DNA fragments found to encode *bla*_CTX–M–55._ The figure shows the different genetic arrangements (lengths) observed around *bla*_CTX–M–55_ encoding DNA fragments illustrated by 6 selected strains **(A)** strain 55A2, **(B)** strain 74A1, **(C)** strains 27C1, **(D)** strain EC297, **(E)** strain 79A1, **(F)** strain 9A2.

The gene *bla*_CTX-M-55_ was the most abundant ESBL-encoding gene observed (*n* = 40 strains). Twenty-eight strains originated from pigs and 12 were from farm workers. In all cases, the *bla*_CTX-M-55_ gene was flanked by an IS*Ecp1* elements upstream and a partial o*rf477* downstream (Figure 1). However, genetic variations between *bla*_CTX-M-55_ encoding elements were frequent due to different length of the IS*Ecp1* elements, different length of the region between IS*Ecp1* and *bla*_CTX-M-55_, and/or different length of the partial o*rf477* (Figure 1). In seven of the 40 strains containing *bla*_CTX-M-55_, BLAST in NCBI suggested a chromosomal location (Table 1 and Supplementary Table S1), as the *bla*_CTX-M-55_ encoding elements were flanked by genes which are normally chromosomally located. In the remaining 33 strains the hosting contigs not only corresponded to plasmid sequences in NCBI (Supplementary Table S1) but also included ORFs which clearly indicated plasmid location (e.g., plasmid DNA primase, DNA ligase, and conserved plasmid hypothetical proteins). The predicted plasmid components carrying *bla*_CTX-M-55_ were of variable replicon types including IncF (IncFIA, IncFIB(K), IncFII), IncX, IncH, IncN, IncR, and IncP (Supplementary File 1). Overall, *bla*_CTX-M-55_ was predominantly plasmid mediated, surrounded by variable genetic contexts and carried on plasmids with multiple different replicon-types. Comparison of isolates obtained from humans and pigs did not suggest transmission of plasmids carrying this gene between pigs and farmers.

**TABLE 1:** Genetic context and location of the main ESBL/AmpC genes detected.

ESBL/AmpC genes	Flanking elements	Plasmid mediated	Chromosome	Plasmid replicons	Selected co-occurring genes
*bla*_CTX–M–55_ (*n* = 40)	IS*Ecp1 and* o*rf477*	33	7	IncF, IncX, IncH, IncN, IncR and IncP	*mcr*-1, *mcr*-3, *qnr*S1, *bla*_*OXA*–10_, *bla*_*TEM*–1B_, *bla*_*LAP*–2_, aadA1, floR, sul2, tet(A), dfrA14
*bla*_CTX–M–27_ (*n* = 36)	IS*Ecp1 and* IS*903B*	36	0	IncF	*bla*_*TEM*–1B_, *bla*_*LAP*–2_, qnrS1, aadA5, aph(3″)-Ib, aph(6)-Id, mph(A), sul1/2, tet(A), dfrA14, dfrA17
*bla*_CTX–M–14_ (*n* = 15)	IS*Ecp1 and* IS*903B*	6	9	IncB/O/K/Z, IncFIB, and IncX4	*bla*_*TEM*–1B_, *bla*_*LAP*–2_, *mcr*-1, *qnr*S1, aac(3)-Iid, cmlA, floR, tet(M), dfrA12
*bla*_CTX–M–15_ (*n* = 10)	IS*Ecp1 and* o*rf477*	9	1	IncB/O/K/Z, IncFII, IncI1	*bla*_*TEM*–1B_, *qnr*S1
*bla*_CTX–M–65_ (*n* = 5)	IS*Ecp1 and* IS*903B*	5	0	IncF	blaTEM-1B, aph(3″)-Ib, aph(6)-Id, sul2
*bla*_CMY–2_ (*n* = 7)	IS*Ecp1 and variable*	2	5	IncF	*bla*_CTX–M–27_, mph(A), aadA5, sul1, dfrA17
*bla*_CMY–42_ (*n* = 1)	IS*Ecp1 and HP*	1	0	IncI1	
*bla*_DHA–1_ (*n* = 2)	IS3E and HP	2	0	IncF	*bla*_CTX–M–27_, qnrB4, mph(A), sul1, dfrA17

#### *bla*_CTX-M-27_

**FIGURE 2:**
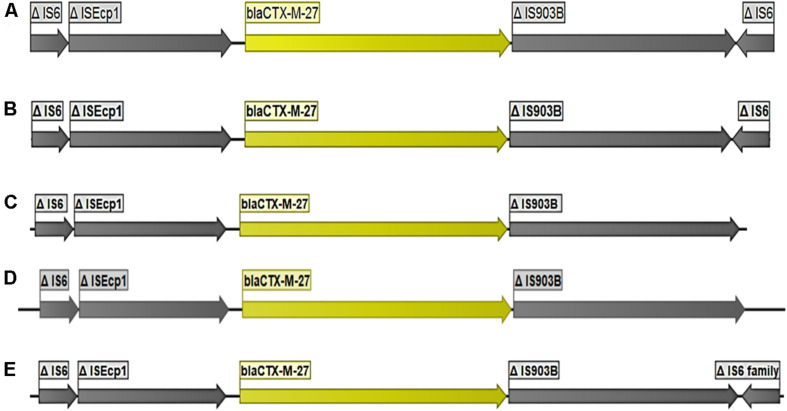
Organization of the DNA fragments found to encode *bla*_CTX–M–27._ The figure represents the consistency in size of elements flanking *bla*_CTX–M–27_ using five isolates: **(A)** strain 90A2, **(B)** strains 8C2, **(C)** strains EC219d), **(D)** strains EC467, **(E)** 100A3.

**FIGURE 3:**
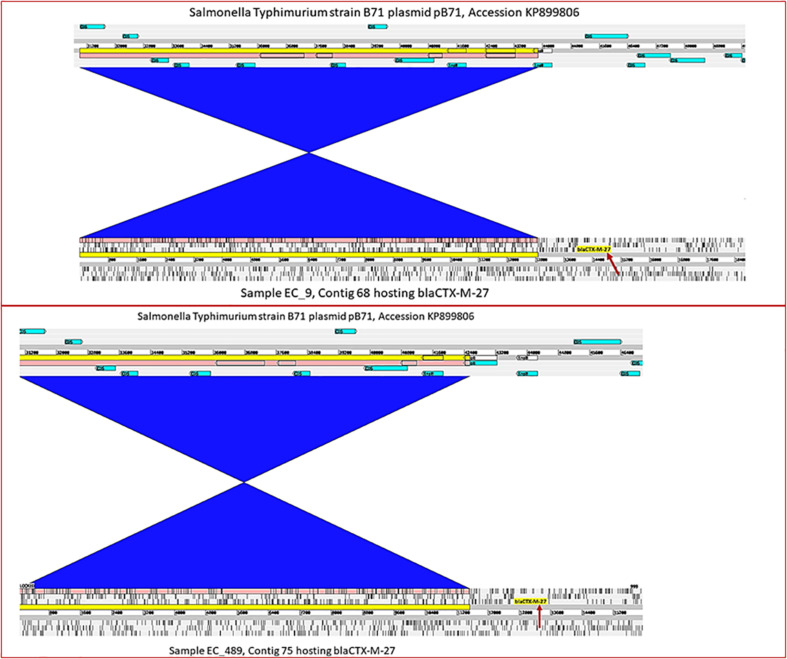
Integrated view of a novel plasmid species harboring *bla*_CTX–M–27_. Representation from strains EC9 and EC489 harboring *bla*_CTX–M–27_ at positions 13997.14886, and 12662.13537, respectively (indicated by the red arrows), in comparison with their closest BLAST reference plasmid pB71 (accession KP899806) which contains no ESBL encoding gene. The blue blocks of synteny indicate an inversion in the shared regions.

The gene *bla*_CTX-M-27_ was detected in 36 *E. coli* strains including 24 strains from pig farm workers and 12 from pigs. *bla*_CTX-M-27_ was flanked in all strains by a partial IS*Ecp1* upstream and partial IS*903B* downstream (Figure 2). In all 36 strains, the contigs where *bla*_CTX-M-27_ was located produced exclusively plasmid hits by BLAST in NCBI, mostly with 99–100% coverage and percent identity match to published plasmid sequences (Table 1 and Supplementary Table S1). The gene *bla*_CTX-M-27_ was associated with IncF conjugative plasmids of variable sizes as confirmed by plasmid profiling (Supplementary File 1). Strikingly, in three strains, we observed that *bla*_CTX-M-27_ was inserted on a novel IncF type of plasmid that had not been reported before (Figure 3). Its closest reference in NCBI was the non-ESBL-encoding *Salmonella* Typhimurium plasmid pB71, Accession KP899806 (70% coverage and 99% identity). *bla*_CTX-M-27_ in ESBL-producing *E. coli* from pigs and pig farm workers were all carried by the conjugal IncF types of plasmid with a consistent genetic context suggesting horizontal transmission of the mobile elements.

#### Other CTX-M Genes

**FIGURE 4:**
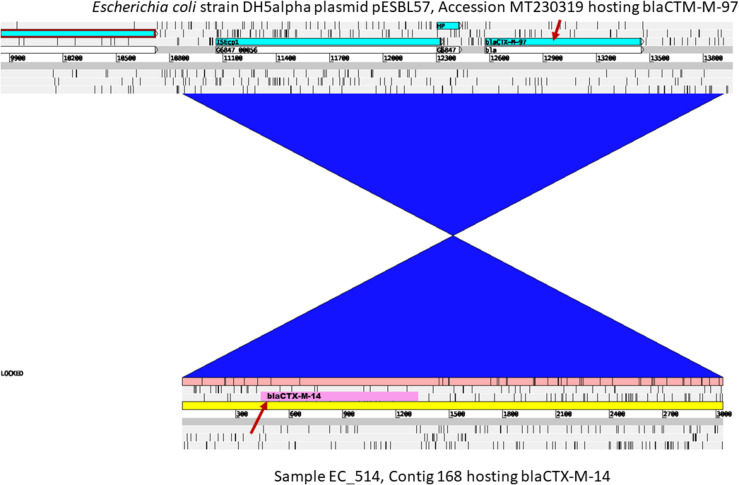
Comparative integrated view of the contig harboring *bla*_CTX–M–14_ in the isolate EC514 vs. plasmid pB71 (accession KP899806). This isolate harbors *bla*_CTX–M–14_ at bp position 466.1341 (indicated by the red arrow below) compared to its closest BLAST reference plasmid pB71 which contains *bla*_CTX–M–97_ (red arrow above). The blue block of synteny indicate an inversion in the shared region.

The gene *bla*_CTX-M-14_ was detected in 15 strains including five strains from humans and ten strains from pigs. *bla*_CTX-M-14_ was flanked by partial IS*903B* interrupted by an IS*Ecp1* upstream and partial IS*903B* downstream (Supplementary Figure S1). This ESBL gene occurred either in the chromosome or on plasmids, i.e., in nine out of the 15 contigs, BLAST showed best hits for *E coli* chromosomal fragments (Table 1 and Supplementary Table S1). In the remaining six strains, the contigs harboring *bla*_CTX-M-14_ showed highly similar nucleotide sequences and perfect matches to plasmid sequences including mostly the *E. coli* plasmid with accession number #MT318677 (Supplementary Table S1). Interestingly, the NCBI BLAST of the *bla*_CTX-M-14_ contig of the human isolate EC514 yielded a perfect match to the *Escherichia coli* plasmid pESBL57, Accession MT230319 initially reported with *bla*_*CTM–*M-97_ (Figure 4). The predicted plasmids harboring *bla*_CTX-M-14_ were of varying sizes as confirmed by profiling (Supplementary File 1) and showed different replicon types including IncB/O/K/Z, IncFIB, and IncX4 (Table 1 and Supplementary Table S1). In summary, *bla*_CTX-M-14_ was predominantly chromosomally located with insertion in variable genetic contexts, and there was little evidence that the gene was transferred between *E. coli* in pigs and farmers.

The gene *bla*_CTX-M-15_ was detected in 10 strains including seven strains from humans and three strains of pig origin. The *bla*_CTX-M-15_ was flanked by an IS*Ecp1* gene upstream and partial *orf477* downstream, which was followed by partial Tn*2* sequence (Supplementary Figure S2). In the human isolate 29C1, *bla*_CTX-M-15_ was predicted to be located in the chromosome (Table 1 and Supplementary Table S1), and annotation of the contig showed that *bla*_CTX-M-15_ and its associated transposon elements were inserted downstream of a 3-dehydroquinate dehydratase annotated as RR31_09005 in *E*. *coli* strain 6409 (GenBank #CP010371). In the remaining nine strains, the *bla*_CTX-M-15_ contig generated plasmid hits in NCBI to which perfect matches (100% coverage and 100%ID) were determined (Supplementary Table S1). The reconstructed plasmids varied in size (Supplementary Table S1 and Supplementary File 1) and were mostly of the IncF conjugal type. Two of them were however multi-replicon plasmids such as in strain 79C1 (IncB/O/K/Z, IncFII), EC472 (IncFII, IncI1), and EC89 (IncFII, IncB/O/K/Z).

The gene *bla*_CTX-M-65_ was present in five strains including three pig strains from 2015, one pig strain from 2018 and one human strain isolated in 2018. Genes in the pig strains were detected on contigs with high similarity (100% coverage and 99.9% identify) to a *Salmonella* Infantis plasmid with accession number CP052840 in NCBI. *bla*_CTX-M-65_ occurred on an IncF conjugal plasmid and in all strains, the *bla*_CTX-M-65_ gene was flanked by insertion sequences *ISEcp1* (IS26) upstream and partial IS*903*-like downstream (Supplementary Figure S3).

One human isolate (EC84 from 2018) carried *bla*_CTX-M-24_ on a 50,655 bp contig. The annotation of the ORFs flancking *bla*_CTX-M-24_ in the contig showed unidentified mobile elements interrupting a tonB dependent receptor upstream and two hypothetical proteins downstream (Supplementary Figure S4A). By BLAST at NCBI, the contig showed the highest similarity (88% coverage and 99.8% ID) with the *E coli* plasmid with the accession number MF136778 (Supplementary Table S1). *bla*_CTX-M-24_ was confirmed on an IncP1 plasmid.

Strain EC488 isolated from a farm worker in 2018 was the only isolate carrying *bla*_CTX-M-3_ on a short 1,289 bp contig that yielded a perfect sequence match by BLAST in NCBI to the *Klebsiella pneumonia* plasmid of accession number LC556222 (Supplementary Table S1). The only annotated ORF on the contig hosting *bla*_CTX-M-3_ was a tryptophan synthase beta chain located downstream (Supplementary Figure S4B). In addition, a 200 kb plasmid of the IncF(II) replicon type was predicted in the isolate and confirmed the plasmid location of *bla*_CTX-M-3_.

#### AmpC Genes

**FIGURE 5:**
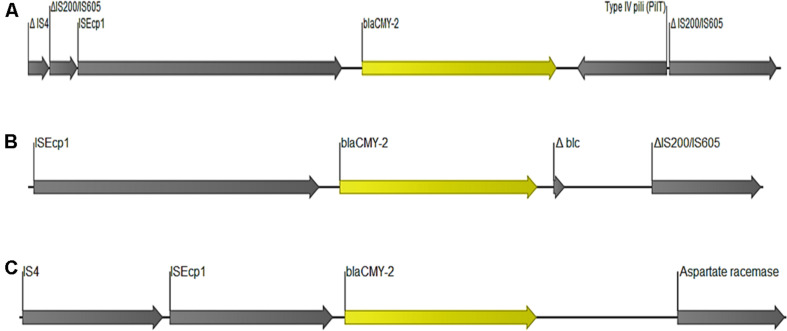
Organization of DNA fragments encoding *bla*_CMY–2_. The annotations depicted here are from isolates **(A)** 31A3, **(B)** 61C2, and **(C)** EC335.

The gene *bla*_CMY-2_ was detected in seven strains. Five of these strains contained the gene in the chromosome (Table 1 and Supplementary Table S1) located together with genes encoding for beta-ketoacyl-ACP synthase, aspartate racemase and transcriptional activator protein LysR. The other two strains carried *bla*_CMY-2_ gene plasmid contig corresponding to *E coli* plasmids #CP034399 and #AP023192 (100% coverage and 99.9%ID). Reconstruction put the gene on IncF conjugal plasmids. In all the seven isolates, *bla*_CMY-2_ was flanked by a complete IS*Ecp1* upstream, whereas the region downstream varied among the strains (Figure 5).

The gene *bla*_CMY-42_ was detected in a single strain from a farm worker on a 33,046 bp contig with IncI1 plasmid elements including *repI1, ardA*, and *trbA*. The *bla*_CMY-42_ gene was detected downstream of a partial IS*Ecp1* and had a hypothetical mobile element downstream (Supplementary Figure S5A). By BLAST at NCBI, this contig showed a perfect match (100% identity and 100% coverage) with *E. coli* plasmid pCMY-42 (KY463221). The reconstructed plasmid from this strain was also of IncI1 replicon type which confirmed the presence of *bla*_CMY-42_ as a plasmid-mediated gene.

The *bla*_DHA-1_ gene was detected in one human (EC495) and one pig strain (EC67). The genetic arrangement of annotated ORFs around *bla*_DHA-1_ in the two strains showed that it was flanked by a transposase *Ins*E for insertion sequence IS3E (upstream) and an undefined mobile element protein downstream (Supplementary Figure S5B). The human isolate harbored *bla*_DHA-1_ on a 145 kb IncFIA plasmid and the pig strain contained the gene on a 170 kb IncF(IB/II) replicon plasmid.

#### Co-occurrence of ESBL/AmpC Encoding Genes With Metal/Biocide, Colistin, and Other Antimicrobial Resistance Genes on Same Plasmids

The plasmid-mediated colistin resistance gene *mcr*-1 was detected in three of the 40 (7.5%) strains from 2015, while 21 of the 74 (28.4%) strains isolated in 2018 carried *mcr*-1 (in combination with *mcr*-3 in four strains). Moreover, multiple co-occurrences were observed, where a chi-square test show a significant association (*p* < 0.018) between the presence of metal/biocide resistance genes and β-lactam genes on the same plasmids (Supplementary Table S1).

For instance, *bla*_CTX-M-55_ co-occurred with the colistin resistance *mcr-1* and *mcr-3* genes along with the quinolone gene *qnrS1* and other β-lactam genes including *bla*_*OXA*-10_, *bla*_*TEM*–1B_, *bla*_*LAP*-2_ as well as other non-ESBL antibiotic resistance genes (Supplementary Table S1). A number of metal and biocide resistance genes also co-occurred with *bla*_CTX-M-55_ on the plasmids including, *marR* (diphenyl ether), *corA* (magnesium-cobalt-nickel-manganese), *ydeP* (hydrochloric acid), *ymgB/ariR* (hydrochloric acid/hydrogen peroxide), *pcoS* (copper), *silA* (silver), *merA* (mercury), *qacEdelta1/qacF/oqxB* (quaternary ammonium compounds) (Supplementary Table S1).

The *bla*_CTX-M-27_ encoding plasmids mostly harbored additional antimicrobial genes including narrow spectrum β-lactamases such as *bla*_*TEM*–1B_ and *bla*_*LAP*-2_ as well as quinolone, aminoglycoside, sulfonamide, and trimethoprim resistance genes (Supplementary Table S1). Moreover, *bla*_CTX-M-27_ plasmids co-carried biocide and metal resistance genes such as *qacEdelta1, emrE/mvrC*, and *qacF* for quaternary ammonium compounds, *zraS* and *zinT* encoding for zinc resistance, *hydG* encoding for lead resistance, *merR* for mercury and the cadmium resistance gene *yod*A (Supplementary Table S1).

Plasmids harboring *bla*_CTX-M-14_, contained various other genes such as *bla*_*TEM*–1B_, *bla*_*LAP*-2_, *mcr*-1, *qnr*S1, and metal resistance genes like *corA* (magnesium, cobalt, nickel, manganese), *mer*A (mercury), *qacEdelta1/qacF* (quaternary ammonium compounds), and *dsbB* (cadmium, mercury) (Supplementary Table S1). *bla*_CTX-M-15_ plasmids harbored *bla*_*TEM*–1B_, *qnr*S1 and biocide resistance genes such as *acrE/envC, qacF* and *qacEdelta1* (Supplementary Table S1). The *bla*_CMY-2_ gene was co-carried with *bla*_CTX-M-27_ and the biocide resistance gene qacEdelta1 encoding resistance to quaternary ammonium compounds (Supplementary Table S1). The *bla*_DHA-1_ gene co-occurred with other antimicrobial genes including notably *bla*_CTX-M-27_, and *qnr*B4 (Supplementary Table S1) and metal/biocide tolerance genes (qacEdelta1, zinT/yodA) with other antimicrobial resistance genes (Supplementary Table S1).

### Diversity and Transferability of Plasmids Harboring the Two Predominant ESBL Genes (*bla*_CTX-M-55_ and *bla*_CTX-M-27_)

Pan-genome analysis of the plasmids harboring *bla*_*CTM–*M-27_ showed that 48 genes out of 1,532 core genes were shared across 80% of the analyzed plasmids. In contrast, only nine genes out of 1,831 constituted the core-genome of plasmids carrying *bla*_*CTM–*M-55_. In both cases, the main core gene was the *bla* gene followed by antimicrobial resistance genes and the plasmid replicon proteins common to the analyzed plasmids.

**FIGURE 6:**
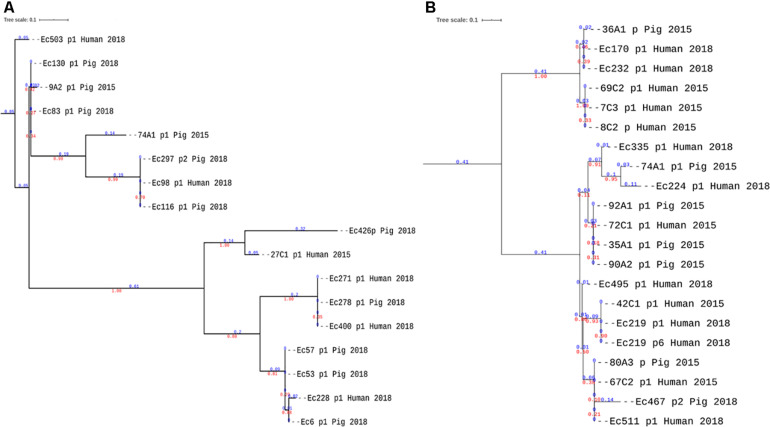
Phylogenetic analyses of reconstructed plasmids carrying *bla*_CTX–M–55_ and *bla*_CTX–M–27_. **(A,B)** Neighbor-joining trees of plasmids harboring *bla*_CTX–M–55_ and *bla*_CTX–M–27_, respectively. The branch lengths are indicated in blue color and the bootstrap values in red. Although plasmids from pigs and farmers are present in the same clades, in panel A of *bla*_CTX–M–55,_ the plasmids are not of the same replicon types (Supplementary Table S1).

The neighbor-joining phylogeny of the alignments of all reconstructed plasmids carrying *bla*_CTX-M-27_ and *bla*_CTX-M-55_ confirmed that plasmids carrying *bla*_CTX-M-55_, showed wider genetic variations with four main clades and longer branch lengths within clades (Figure 6A) compared to the more homogenous IncF *bla*_CTX-M-27_ plasmids that was found in pigs and farmers (Figure 6B). Although some *bla*_CTX-M-55_ plasmids from pigs and farmers clustered in the same clades (Figure 6A), these plasmids were not of the same replicon types and therefore did not provide evidence of transmission between hosts, but rather supported that this gene is carried by different plasmids with different transposons.

The plasmids carrying *bla*_CTX-M-27_ and *bla*_CTX-M-55_ were confirmed as conjugative with transfers frequencies varying from 6.55 × 10^-^^6^ to 3.06 × 10^-^^4^ with a slight increase when the conjugation time increased (Table 2). Moreover, in the strains with multiple plasmids subjected to the conjugation experiment, only plasmids encoding the *bla*_CTX-M_ gene were transferred to the transconjugants (Supplementary Figure S6).

**TABLE 2:** Conjugation transfer frequency of *bla*_CTX–M–27_ and *bla*_CTX–M–55_ encoding plasmids.

Donors	Conjugation time	CFU/mL of donors (CTX plates)	CFU/mL of transconjugants (CTX + RIF plates)	Conjugation transfer frequency (%)
EC224 (IncFII/*_*bla*_*_CTX–M–27_)	1 h	2.43 × 10^10^ ± 0.48 × 10^10^	2.48 × 10^2^ ± 0.56 × 10^2^	1.02 × 10^–6^
	6 h	2.74 × 10^10^ ± 0.58 × 10^10^	1.26 × 10^3^ ± 0.25 × 10^3^	4.59 × 10^–6^
EC297 (IncFIB/*_*bla*_*_CTX–M–55_)	1 h	2.47 × 10^10^ ± 0.71 × 10^10^	5.60 × 10^2^ ± 1.27 × 10^2^	2.27 × 10^–6^
	6 h	2.42 × 10^10^ ± 0.46 × 10^10^	1.58 × 10^3^ ± 0.25 × 10^3^	6.55 × 10^–6^
EC170 (IncFIB/*_*bla*_*_CTX–M–27_)	1 h	2.55 × 10^10^ ± 0.50 × 10^10^	5.83 × 10^3^ ± 0.67 × 10^3^	2.28 × 10^–5^
	6 h	1.55 × 10^10^ ± 0.47 × 10^10^	5.48 × 10^4^ ± 0.85 × 10^4^	3.53 × 10^–4^
EC116 (IncN/*_*bla*_*_CTX–M–55_)	1 h	2.26 × 10^10^ ± 0.56 × 10^10^	5.05 × 10^3^ ± 0.57 × 10^3^	2.24 × 10^–5^
	6 h	2.15 × 10^10^ ± 0.23 × 10^10^	6.58 × 10^4^ ± 0.83 × 10^3^	3.06 × 10^–4^

### *E. coli* Diversity

**FIGURE 7:**
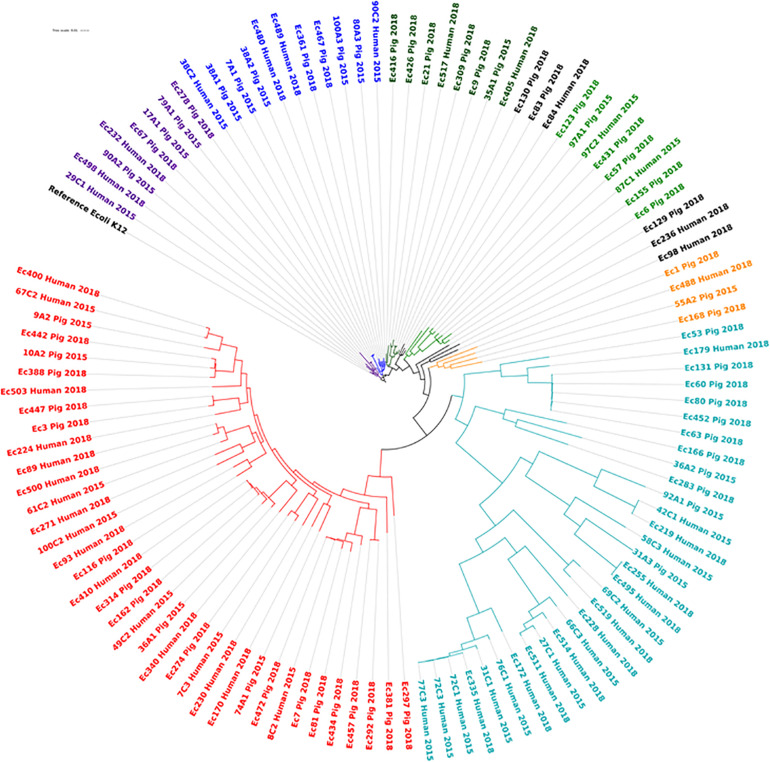
Phylogenetic diversity of ESBL/*Amp*C producing *E. coli* isolated from pig and pig farm workers in Northern Vietnam in 2015 and 2018. Each phylogenetic clade is indicated with a separate color. Although isolates from pigs and farmers are present in the same clades, the SNP values between them show wide genetic variations and they are of different ST types (Supplementary Table S2).

Of the 114 *E. coli* strains analyzed, 82 different sequence types were detected (Supplementary Table S2, sheet 2) but only three sequence types were shared between human and pig isolates. These include ST10 reported in 10 pig isolates and in 1 human isolate, while ST48 and ST2170 were found in one pig and 1 animal isolate, respectively. None of these few-shared STs was from the same farms. The core genome phylogenetic analysis of the 113 isolates against the reference *E. coli* K12 confirmed the wide genetic variation among the isolates regardless of host, the farm and years of isolation with up to 39,306 SNPs difference between strains grouped in nine clades (Figure 7). None of the STs was predominantly driving a specific plasmid or ESBL encoding gene. In all of these clades, there were isolates from both pig and farm workers, however the isolates were often of different sequence types with wide SNPs differences (Supplementary Table S2). The strains were predicted to be of commensal types, since their virulence profiles did not correspond to main *E. coli* pathotypes (Supplementary Table S1, sheet 2).

## Discussion

It is widely suggested that the livestock sector may be an important source of antimicrobial resistance genes, including ESBL/AmpC encoding genes, found in humans. The transfer mainly happens through horizontal spread of mobile elements (Zaja̧c et al., 2019; Dantas Palmeira and Ferreira, 2020). To elucidate the level of transmission of ESBL/AmpC genes in *E. coli* from pig and pig farmers in Vietnam, we performed a detailed genetic analysis of *E. coli* strains, which harbored such genes, and we analyzed the location of genes in the genome. This included annotation of transposons and insertion sequences located around the resistance genes, the phylogenetic relatedness of the plasmid components reconstructed from strains, and genetic relatedness of the strains, all based on whole genome sequences. This approach provides a holistic answer to the transmission of resistance genes between hosts, either by transfer of strains or by spread of mobile genetic elements, and thus it extends the approaches used in most previous studies of spread of resistance genes and mobile genetic element between hosts (Kudirkiene et al., 2018; Zaja̧c et al., 2019; Baniga et al., 2020). Analysis of the plasmids and the genetic contexts along with the strains diversity allow to investigate the diversity, distribution, and transmission dynamics of resistance plasmids in the strains (David et al., 2020). The ESBL/AmpC β-lactamases genes detected in the analyzed strain collection of *E. coli* were present across pigs and pig farmers suggesting a distribution of the same genes across hosts, however, the most commonly observed gene differed between the two hosts; *bla*_CTX-M-55_ was the most commonly observed among the pig isolates and *bla*_CTX-M-27_ among human isolates. The genes detected have previously been described from diseased and healthy humans, chickens, pigs, and food products in Vietnam in studies where the human cases were suggested to emanate from animal origins (Nguyen et al., 2016; Hoang et al., 2017; Hinenoya et al., 2018).

The predominant ESBL gene, *bla*_CTX-M-55_ was mainly plasmid mediated, and was found to be carried on different types of conjugative plasmids with highly variable genetic contexts around the ESBL genes, just as the insertion transposases downstream of the gene were also mostly different. Previous reports have found *bla*_CTX-M-55_ on chromosomal fragments as well as on IncHI2 (Zhang et al., 2019), Incl2 (Lv et al., 2013), and IncF and Incl2 (Lupo et al., 2018) plasmids. The diversity observed in types of plasmids suggested limited transmission *bla*_CTX-M-55_ between pigs and farm workers. The phylogenetic analyses of the plasmids carrying *bla*_CTX-M-55_ supported that plasmids in strains from pigs and humans were unrelated, e.g., different replicon types were found in the two hosts. Another supporting observation was the fact that the plasmids did not share reference plasmids in NCBI to any large extend. For instance, no more than five contigs with *bla*_CTX-M-55_ showed similarity to a common plasmid reference in the database [plasmid accessions MN823991 (5), AP023198 (5), MN158989 (5), CP034747 (4)]. Overall, these findings with regard to plasmids carrying *bla*_CTX-M-55_, as well as plasmid carrying *bla*_CTX-M-14_, *bla*_CTX-M-15_, and *bla*_CMY-2_ indicated that horizontal gene transfer in a real-life situation between pigs and pig farm workers happens with so low frequency that it will only be detected by very intensive sampling strategies. However, laboratory-based conjugation experiments shows that transfer of ESBL-encoding plasmids between *E. coli* strains is more frequent (Liu et al., 2019). Plasmids harboring *bla*_CTX-M-55_ were confirmed to be conjugative in our investigation with similar conjugation transfer frequencies as plasmids encoding *bla*_CTX-M-27_. Whether transferability of plasmids between strains under field conditions is as efficient as in the laboratory remains to be investigated. The presence of *bla*_CTX-M-55_ on diverse plasmid replicon types with different transposons on various *E. coli* lineages show the heterogeneity of the plasmids carrying this ESBL gene, and suggest multiple introductions of these genes independently in each host (David et al., 2020). Together with the high variation in genetic context around the *bla*_CTX-M-55_ gene, these results show that *bla*_CTX-M-55_ in farmers is not necessarily horizontally acquired from close contact with animals and further that there is limited transmission of this type of resistant *E. coli* from pigs to farmers, and *vice versa*. The sample size is, however, not large enough to rule out that transmission occurs on a low level, given the fact that the plasmids are transferable.

The gene *bla*_CTX-M-27_, was the second leading ESBL gene found in pigs and pig farmers. It was located in a relatively conserved genetic context in all isolates with the same flanking transposons in all the strains and all carried by the same conjugal IncF types of plasmid. The strains carrying the gene, on the other hand did not show resemblance. This is evidence of horizontal spread of the IncF plasmids types carrying *bla*_CTX-M-27_ in the study area. The same types of conjugative IncF plasmids were found in *E. coli* strains from pigs and farm workers (Supplementary Table S1), however, the direction of transfer of plasmids cannot be established from the current study. The gene *bla*_CTX-M-27_ seems to be generally associated with conjugative plasmids of the IncF replicon type including FIA, FIB, and FII (Fernandes et al., 2020; Matsuo et al., 2020). The phylogenetic analysis of the plasmids revealed that they shared a larger core-genome cluster compared to the plasmids harboring *bla*_CTX-M-55_. In contrast to the observation with the plasmids carrying *bla*_CTX-M-55_, many of the *bla*_CTX-M-27_ plasmids shared reference plasmids in NCBI (plasmid accession CP049168 shared by 11 strains and KX008967 by nine strains), further supporting the low genetic variation for this type of plasmids. We identified a novel plasmid species harboring *bla*_CTX-M-27_ in three strains. Its closest reference in NCBI, *Salmonella* Typhimurium plasmid pB71, is a non-ESBL, multi-drug resistance plasmid, and it is likely that it has experienced an insertion of *bla*_CTX-M-27_, however, other ways including intermediate plasmid species, could also have led to the formation of this plasmid. The size of the plasmid was approximately 150 kb (Supplementary Table S1 and Supplementary File 1). It is an IncF replicon type plasmid, and encoded multi drug resistance with *mcr*-3, *aad*A1, *aac*(3)-Iid, *aph*(3″)-Ib, *aph*(3′)-Ia, *aph*(6)-Id, *mef*(B), *cml*A1, *flo*R, *sul*3, and *dfr*A12 genes in addition to the ESBL gene. Overall, this novel plasmid is similar to the other *bla*_CTX-M-27_ plasmid reported in this study and elsewhere (Fernandes et al., 2020; Matsuo et al., 2020).

Moreover, our comparative analyses of contigs carrying the IncF types of plasmids also revealed another plasmid that showed complete identity with *E. coli* plasmid pESBL57, Accession MT230319 initially harboring *bla*_*CTM–*M-97_ in NCBI. Our analysis indicates that *bla*_CTX-M-14_ has replaced *bla*_*CTM–*M-97_.

Five of the seven strains where *bla*_CMY-2_ was detected carried the gene in the chromosome. The other AmpC genes detected, *bla*_CMY-42_ and *bla*_DHA-1_, were plasmid-mediated with consistent genetic contexts between isolates from pigs and humans, indicating that these genes are transmitted between hosts. In other studies, *bla*_CMY-2_ was reported to be chromosomally located (Harada et al., 2010), which suggests that this gene is less likely mobilizable and can only be spread together with the host strains. In many cases, the plasmids containing ESBL/Ampc β-lactamases also carried *mcr*-1 or *mcr-*3 genes encoding colistin resistance, as well as the presence of metal/biocide tolerance genes. Co-occurrence of ESBL/AmpC, colistin resistance as well as other antimicrobial resistance genes such as *qnrS1, mef(B), mph(A), cmlA1, sul1, sul3, tet(A), dfrA12, aadA1/2, aac(3)-IIa* was common in most isolates, and resistance genes were often occurring on the same plasmid (Supplementary Table S1). This corroborate previous resports that the majority of the strains analyses had multidrug resistant status (Dang et al., 2018). The plasmids identified therefore represent an important source of multidrug resistance, and due to their location on conjugative plasmids, they may transfer resistances of significant public health importance, and such plasmids need particular attention to track their global emergence, evolution and spread (Lupo et al., 2018; Zaja̧c et al., 2019). Our findings further corroborate studies showing co-carriage of *mcr-*genes in many ESBL isolates (Lupo et al., 2018; Zaja̧c et al., 2019) although, in those studies, the *mcr*-encoding plasmids are not the same as the plasmid carrying the ESBL genes.

In our study, ESBL/AmpC and other antimicrobial resistance genes were frequently co-located with heavy metal resistance genes on the same plasmids. Heavy metal exposure due to usage in livestock feed, e.g., use of zinc oxide in pig feed, can therefore co-select for antimicrobial resistance in bacteria (Ludden et al., 2019; Peng et al., 2020). In support of this, recent studies have reported that antimicrobial resistance is associated with tolerance to heavy metals existing naturally or used in food animal production, including zinc oxide and copper (Rensing et al., 2018; Cheng et al., 2019; García et al., 2020). Studies have also reported occurrence of metal tolerance genes in *E. coli* from post-weaning diarrhea in piglets with no documented exposure to metals, probably reflecting that over the years, metal resistance genes have been widely spread among *E. coli* in pigs (García et al., 2020).

The phylogenetic analysis of the isolates revealed wide genetic variation with no evidence of transmission of strains between pigs and farmers, and vice versa. All the isolates were of different MLST types, and none of them was predicted to be of known pathogenic sub-types (Supplementary Table S1). Thus, ESBL and AmpC encoding *E. coli* in pigs and pig farmers in Vietnam belong to unrelated commensal groups with both related and unrelated plasmids. A recent study has shown that *E. coli* causing bloodstream infections in the United Kingdom were not acquired from livestock, and that sharing of mobile elements between animals and humans was infrequent (Ludden et al., 2019). Moreover, a large diversity was reported in *E. coli* clones and plasmid types in France from food animals corroborating our findings (Lupo et al., 2018). Thus, our findings provide further evidence that plasmids carrying ESBL/AmpC β-lactamase encoding genes occur in genetically unrelated commensal *E. coli*. Strain sharing between the two hosts was infrequent and so was the mobile genetic element except for *bla*_CTX-M-27_, and this underlines that one cannot only focus on the resistant strains when analyzing spread patterns of resistance genes.

Despite that the bacterial strains from 2015 (Dang et al., 2018) and 2018 were epidemiologically related, i.e., matching fecal samples from workers and pigs collected from the same pig farms, they showed genetic variations and were predominantly commensals occurring independently in each host. Moreover, commensal *E. coli* are less studied than pathogenic *E. coli* with the former usually found genetically distinct (Ahmed et al., 2017). This may hide the occurrence of frequent transfer of mobile elements across strains as suggested by the conjugation experiments. Furthermore, as mobilizable genes, ESBL carriage is dynamic and could affect the consistency of their contexts in cross-sectional studies. However, the fact that sampling at two separate time frames did not show any significant fluctuations in the genetic context of ESBL genes in pigs and farm workers suggests that most of the ESBL genes are less frequently shared between the two hosts. Nevertheless, future longitudinal studies would provide further and improved insights.

A main limitation in this study is that only short-read technologies were used to generate the whole genome and plasmid sequences, Long and short-reads hybrid assemblies do not show significantly different outcomes (David et al., 2020), however, using combinations of the two techniques, closing of circular (plasmid) sequences is possible. To overcome the limitation, we used plasmid profiling to confirm the results of the predicted plasmids and their sizes, and conjugation experiment were further used to confirm the transferability of the plasmids between strains. We suggest that the relatedness of the plasmids encoding the resistances and the consistency of the genetic context around the ESBL/Ampc genes is considered along with the phylogenetic relationship between the strains.

## Data Availability Statement

The datasets presented in this study can be found in online repositories. The names of the repository/repositories and accession number(s) can be found in the article/Supplementary Material.

## Author Contributions

AD and JO: conceptualization and supervision. YH and VB: methodology and software and data curation. YH, VB, AD, JO, SD, and DT: validation and writing—review and editing. YH: formal analysis, writing—original draft preparation and visualization. YH, SD, and DT: investigation. YH, VB, AD, and JO: resources. AD: project administration and funding acquisition. All authors contributed to the article and approved the submitted version.

## Conflict of Interest

The authors declare that the research was conducted in the absence of any commercial or financial relationships that could be construed as a potential conflict of interest

## Footnotes

1 http://resistancecontrol.info/2017/the-esbl-tricycle-amr-surveillance-project-a-simple-one-health-approach-to-global-surveillance/

2 www.random.org

3 http://www.genomicepidemiology.org/

4 https://www.ebi.ac.uk/Tools/msa/clustalo/

## Supplementary Material

Supplementary File 1 Gel images of the plasmid profiling results.

Supplementary Figure 1 Organization of the gene fragments encoding *bla*_CTX-M-14._ The figure represents the different genetic arrangements of *bla*_CTX-M-14_ in the isolates containing the gene with the lengths of the different insertion sequences flanking the *bla* gene. The isolates displayed here are **(A)** 38C2, **(B)** 87C1, and **(C)** EC172.

Supplementary Figure 2 Organization of the DNA fragments encoding *bla*_CTX-M-15._ The figure represents the different genetic arrangements of *bla*_CTX-M-15_ in the isolates harboring the gene with the lengths of the different insertion sequences flanking the *bla* gene. The isolates displayed here are **(A)** 17A1, **(B)** 76C1, and **(C)** EC472.

Supplementary Figure 3 Organization of the DNA fragments encoding *bla*_CTX-M-65._ The figure represents the different genetic arrangements of *bla*_CTX-M-65_ in the isolates harboring the gene with the lengths of the different insertion sequences flanking the *bla* gene. The isolates displayed here are **(A)** 7A1, **(B)** 38A1, and **(C)** EC93.

Supplementary Figure 4 Organization of the DNA fragments encoding *bla*_CTX-M-24_ and *bla*_CTX-M-3._ The annotations depicted here are from isolates **(A)** EC84 and **(B)** EC488.

Supplementary Figure 5 Organization of the DNA fragments encoding *bla*_CMY-42_ and *bla*_DHA-1._ The annotations depicted here are from isolates **(A)** 100C2 and **(B)** EC495.

Supplementary Figure 6 Plasmid profile of transconjugant strains after conjugation. The samples M1 and M2 are the two reference strains 39R861 and V517, respectively, serving as size markers. R is the recipient strain *E. coli* J53-1. D1: is the donor EC224, D2: EC297, D3: EC170, D4: EC116. T stands for transconjugants.

Supplementary Table 1 Overall characterization of the ESBL-producing *E. coli* genomes.

Supplementary Table 2 Pairwise SNPs between ESBL-producing *E. coli* genomes.
