# Supplementary material for: ESBL and AmpC β-Lactamase Encoding Genes in E. coli From Pig and Pig Farm Workers in Vietnam and Their Association With Mobile Genetic Elements
Source: Front Microbiol. 2021 Mar 11;12:629139. doi: 10.3389/fmicb.2021.629139 (PMC7991805; doi:10.3389/fmicb.2021.629139)

**Supplementary file 1: Plasmid Profiling Figures**

All the gel images below are labelled in PowerPoint with the samples ID in the displayed order. The samples M1 and M2 are the two reference strains 39R861 and V517 respectively and served as markers.


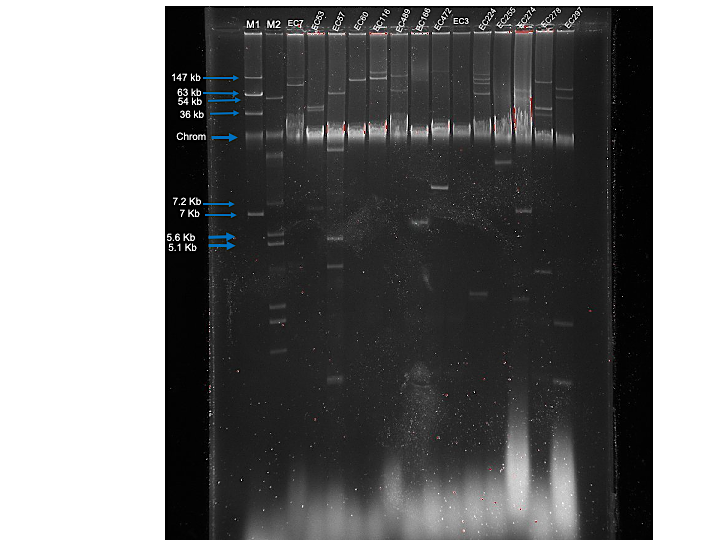


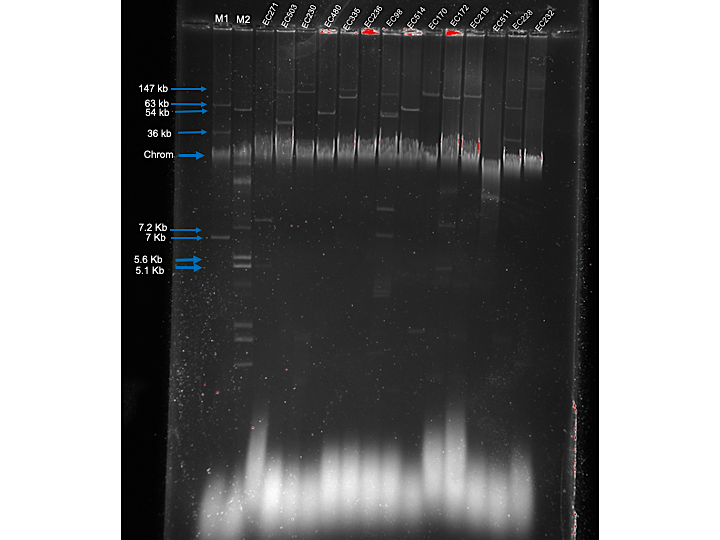


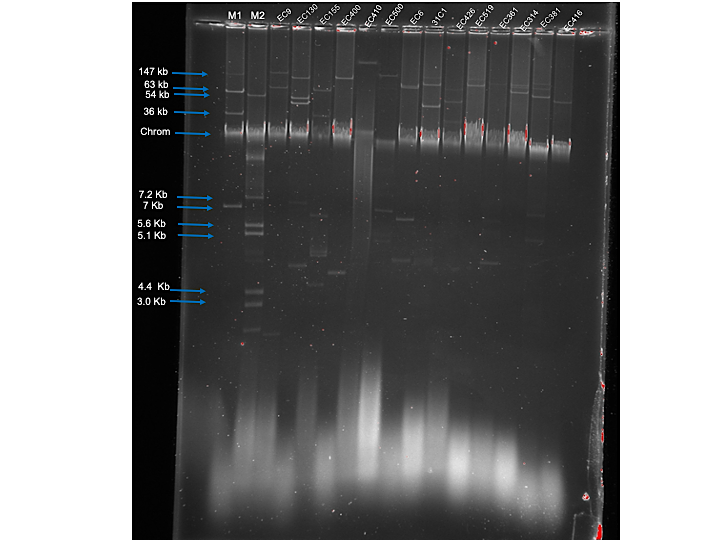


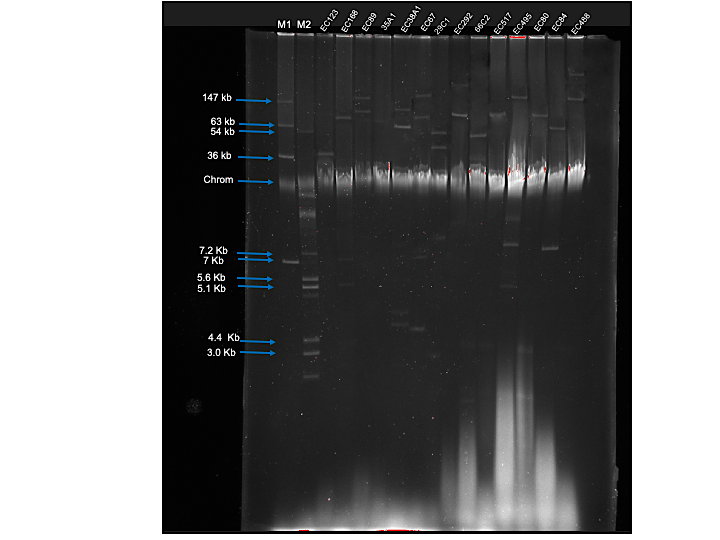

Supplement: Supplementary File 1 — Gel images of the plasmid profiling results. [file Data_Sheet_1.docx]
