# Supplementary figures and images for: ESBL and AmpC β-Lactamase Encoding Genes in E. coli From Pig and Pig Farm Workers in Vietnam and Their Association With Mobile Genetic Elements
Source: Front Microbiol. 2021 Mar 11;12:629139. doi: 10.3389/fmicb.2021.629139 (PMC7991805; doi:10.3389/fmicb.2021.629139)

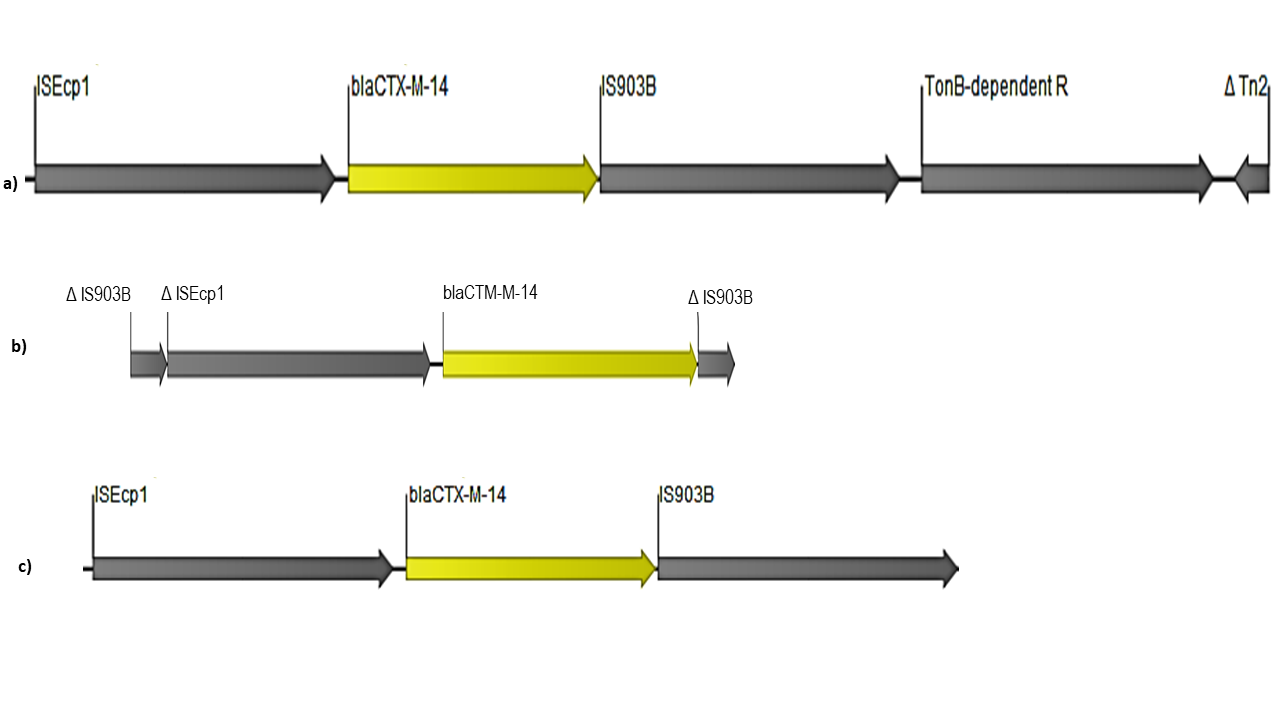

Supplement: Supplementary Figure 1 — Organization of the gene fragments encoding blaCTX-M-14. The figure represents the different genetic arrangements of blaCTX-M-14 in the isolates containing the gene with the lengths of the different insertion sequences flanking the bla gene. The isolates displayed here are (A) 38C2, (B) 87C1, and (C) EC172. [file Image_1.TIF]

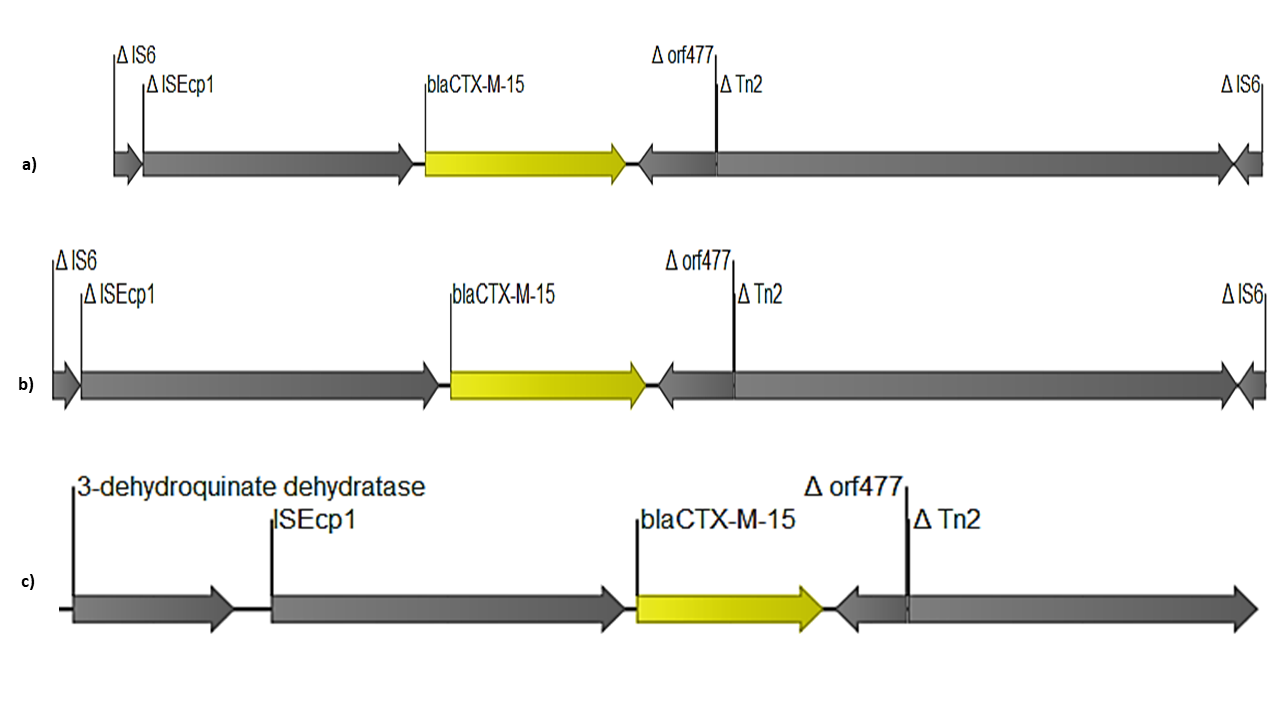

Supplement: Supplementary Figure 2 — Organization of the DNA fragments encoding blaCTX-M-15. The figure represents the different genetic arrangements of blaCTX-M-15 in the isolates harboring the gene with the lengths of the different insertion sequences flanking the bla gene. The isolates displayed here are (A) 17A1, (B) 76C1, and (C) EC472. [file Image_2.TIF]

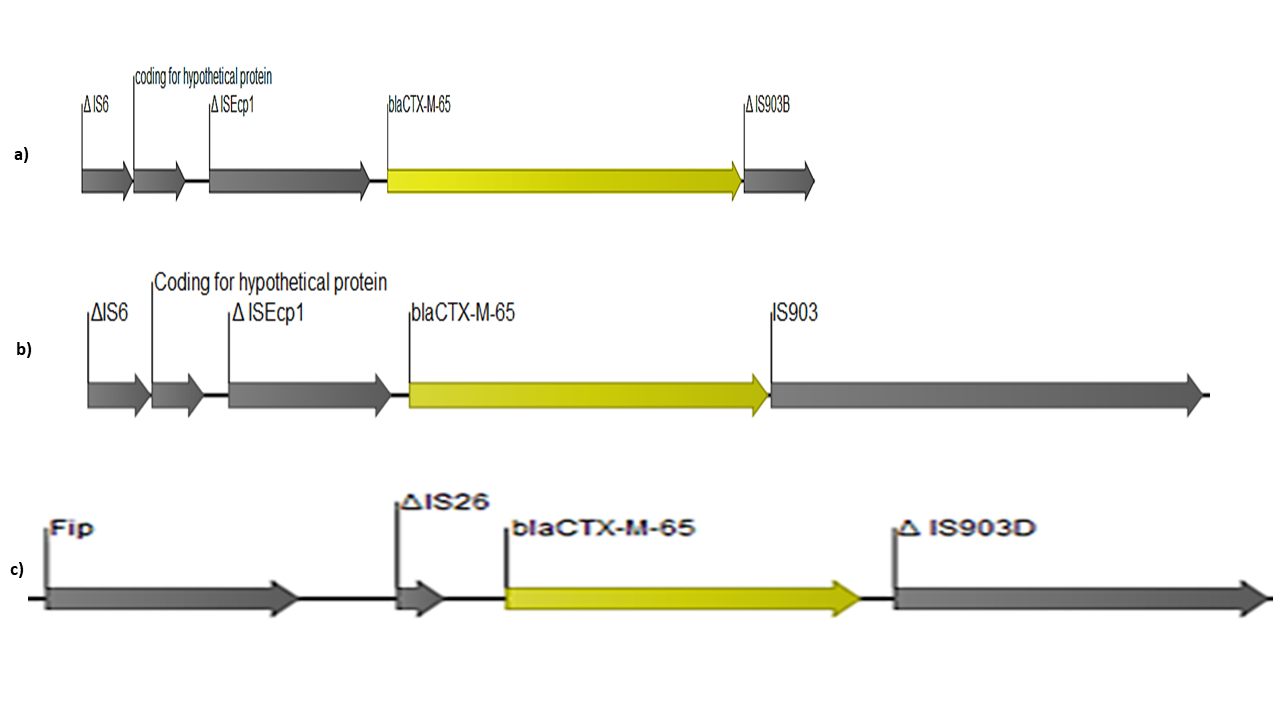

Supplement: Supplementary Figure 3 — Organization of the DNA fragments encoding blaCTX-M-65. The figure represents the different genetic arrangements of blaCTX-M-65 in the isolates harboring the gene with the lengths of the different insertion sequences flanking the bla gene. The isolates displayed here are (A) 7A1, (B) 38A1, and (C) EC93. [file Image_3.TIF]

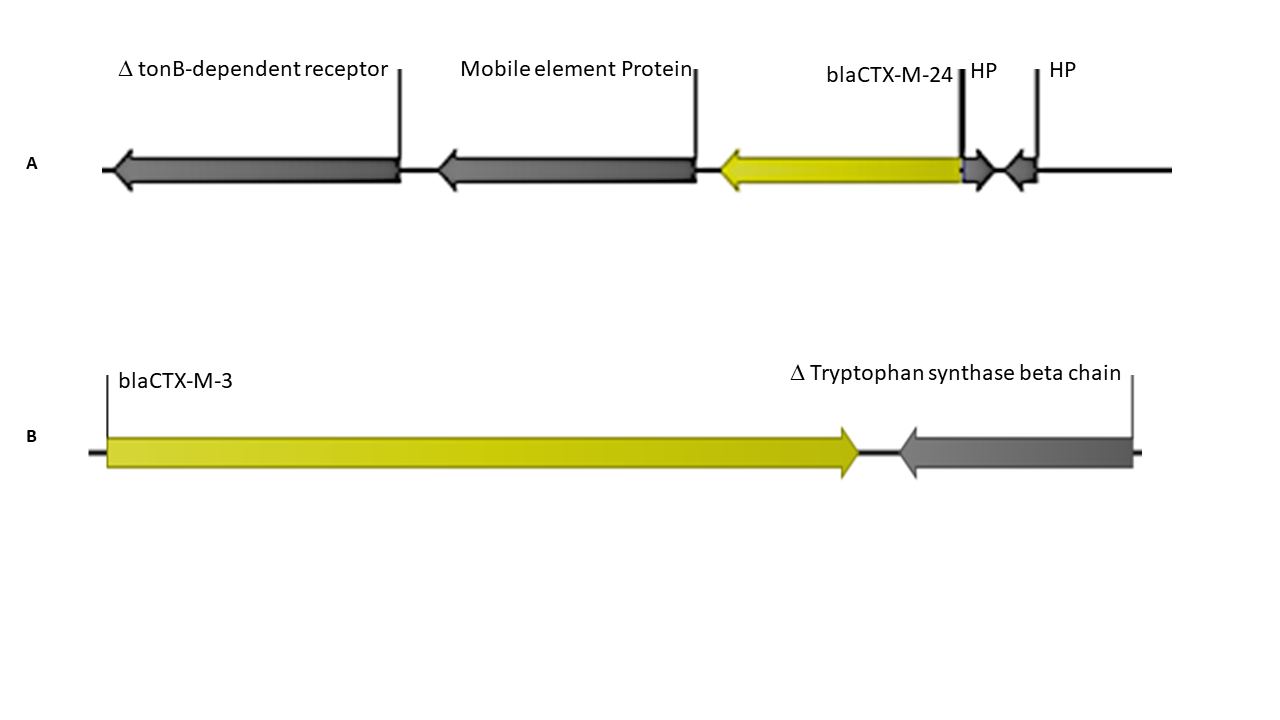

Supplement: Supplementary Figure 4 — Organization of the DNA fragments encoding blaCTX-M-24 and blaCTX-M-3. The annotations depicted here are from isolates (A) EC84 and (B) EC488. [file Image_4.TIF]

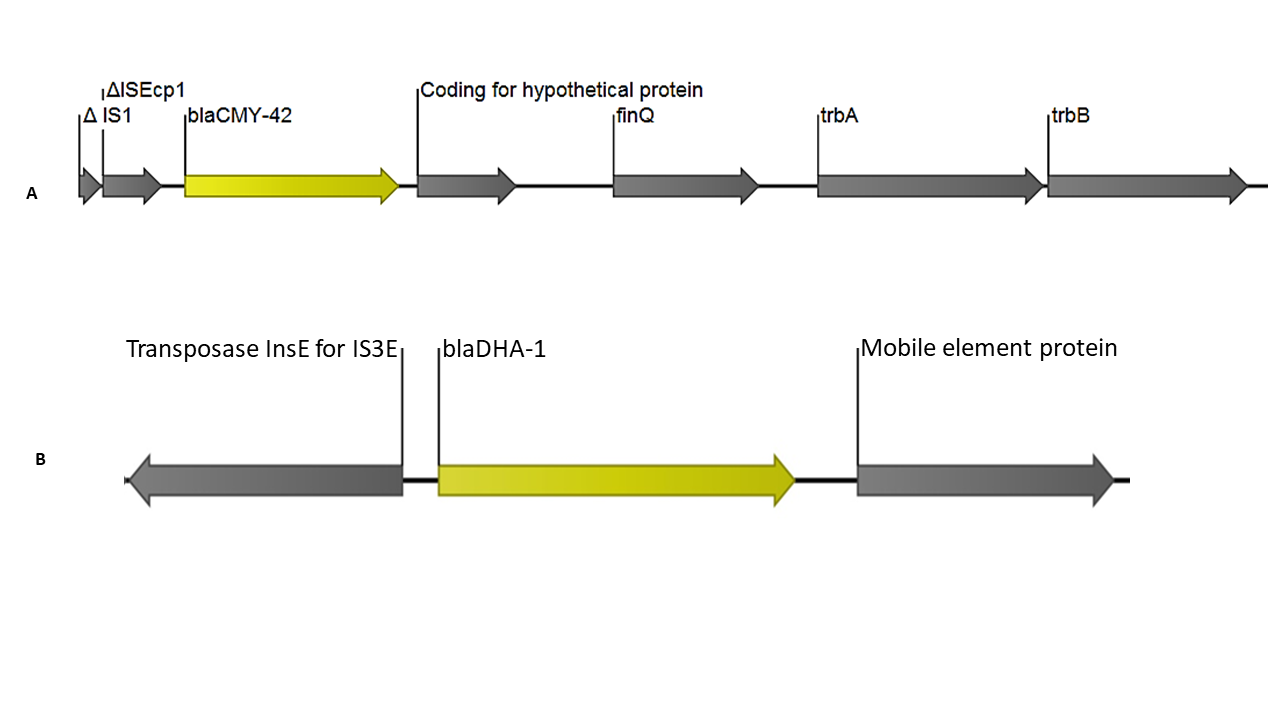

Supplement: Supplementary Figure 5 — Organization of the DNA fragments encoding blaCMY-42 and blaDHA-1. The annotations depicted here are from isolates (A) 100C2 and (B) EC495. [file Image_5.TIF]

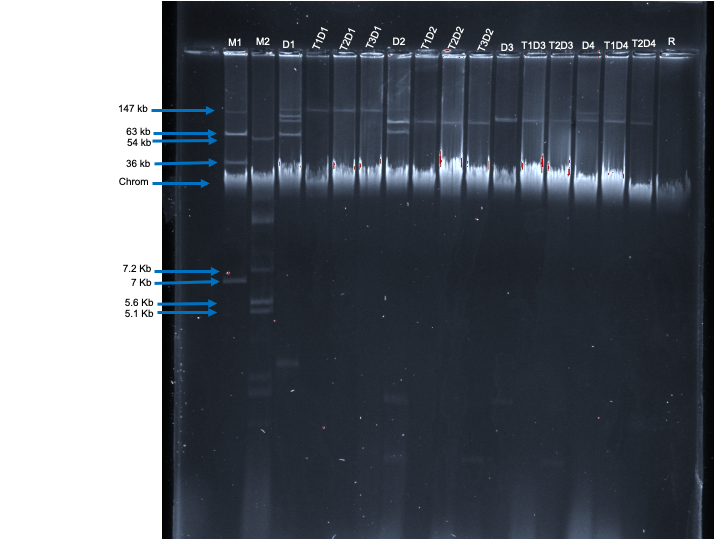

Supplement: Supplementary Figure 6 — Plasmid profile of transconjugant strains after conjugation. The samples M1 and M2 are the two reference strains 39R861 and V517, respectively, serving as size markers. R is the recipient strain E. coli J53-1. D1: is the donor EC224, D2: EC297, D3: EC170, D4: EC116. T stands for transconjugants. [file Image_6.TIFF]
